# SIAH2–WNK1 Signaling Drives Glycolytic Metabolism and Therapeutic Resistance in Colorectal Cancer

**DOI:** 10.3390/ijms27021065

**Published:** 2026-01-21

**Authors:** Kee-Thai Kiu, Cheng-Ying Chu, Yi-Chiao Cheng, Min-Hsuan Yen, Ying-Wei Chen, Narpati Wesa Pikatan, Vijesh Kumar Yadav, Tung-Cheng Chang

**Affiliations:** 1 Division of Colorectal Surgery, Department of Surgery, Taipei Medical University Shuang-Ho Hospital, New Taipei City 235, Taiwan; kiubabar@gmail.com (K.-T.K.); 17251@s.tmu.edu.tw (M.-H.Y.); 16402@s.tmu.edu.tw (Y.-W.C.); vijeshp2@gmail.com (V.K.Y.); 2 Division of General Surgery, Department of Surgery, School of Medicine, College of Medicine, Taipei Medical University, Taipei 110, Taiwan; 3 CRISPR Gene Targeting Core, Taipei Medical University, Taipei 110, Taiwan; cchu@tmu.edu.tw; 4 TMU Research Center of Cancer Translational Medicine, Taipei Medical University, Taipei 110, Taiwan; 5 Division of Colon and Rectal Surgery, Department of Surgery, Tri-Service General Hospital, National Defense Medical University, Taipei 114, Taiwan; ndmcjoe@gmail.com; 6 Division of Urology, Department of Surgery, Faculty of Medicine, Universitas Gadjah Mada, Yogyakarta 55281, Indonesia; narpatiwp@gmail.com

**Keywords:** metastatic colorectal cancer stem cells, tumor fibroblasts, SIAH2/WNK1 signaling

## Abstract

Colorectal cancer (CRC) progression and therapy resistance are driven in part by metabolic reprogramming and the persistence of cancer stem-like cells (CSCs). The seven in absentia homolog 2 (SIAH2)/with-no-lysine kinase 1 (WNK1) signaling axis has emerged as a potential regulator of these processes, yet its functional role in CRC metabolism and tumor–stroma crosstalk remains incompletely understood. Integrated analyses of The Cancer Genome Atlas–Colon Adenocarcinoma (TCGA-COAD) and Gene Expression Omnibus (GEO, GSE17538) datasets revealed significant upregulation of *SIAH2* and *WNK1* in CRC tissues, with strong positive correlations to glycolysis- and hypoxia-associated genes, including *PFKP*, *LDHA*, *BPGM*, *ADH1A, ADH1B,* and *HIF-1α*. Single-cell and clinical profiling further demonstrated preferential enrichment of *SIAH2* in undifferentiated, stem-like tumor cell populations. Functional studies across multiple CRC cell lines showed that *SIAH2* silencing suppressed proliferation, clonogenic growth, tumor sphere formation, and cell-cycle progression, whereas *SIAH2* overexpression exerted opposite effects. Seahorse extracellular flux analyses established that *SIAH2* promotes glycolytic capacity and metabolic flexibility. At the protein level, SIAH2 regulated glycolytic enzymes and WNK1/hypoxia-inducible factor-1α (HIF-1α) signaling, effects that were amplified by cancer-associated fibroblast (CAF)-derived conditioned medium. CAF exposure enhanced SIAH2 expression, CSC spheroid growth, and resistance to fluorouracil, leucovorin, and oxaliplatin (FOLFOX) chemotherapy, whereas *SIAH2* depletion effectively abrogated these effects. Collectively, these findings identify the SIAH2/WNK1 axis as a central metabolic regulator linking glycolysis, CSC maintenance, and microenvironment-driven therapy resistance in CRC, highlighting its potential as a therapeutic target.

## 1. Background

Colorectal cancer (CRC) is currently the third leading cause of cancer-related deaths in Taiwan and has the highest annual incidence of new cases among all cancers in the country [1]. While early-stage CRC can often be effectively treated with surgery, leading to favorable outcomes, advanced stages of the disease frequently involve metastasis to lymph nodes and distant organs, where combination chemotherapy serves as the primary treatment [2]. However, the 5-year survival rate for advanced CRC remains distressingly low, below 10%. This grim statistic underscores the urgent need for more effective treatments, especially for advanced and challenging cases of CRC [3]. Recent research has spotlighted a small subset of cells known as colon cancer initiating cells (CCICs), which possess self-renewal capabilities, sustain the tumor, and contribute to distant metastases [4]. Targeting and eradicating these CCICs has become a pivotal focus in CRC treatment and drug development. The prognosis for CRC is often unfavorable due to high rates of both local and distant metastasis and recurrence. While patients with localized CRC have a 5-year survival rate of around 90%, this rate drops drastically to only 10–20% for those with metastatic disease [5]. Cancer stem cells (CSCs), a subset of primary CRC cells, are largely responsible for chemoresistance and recurrence due to their high tumorigenic potential and ability to self-renew [6]. Despite extensive research, the fundamental mechanisms driving metastasis, relapse, and mortality in CRC remain elusive. This complexity is further compounded by the characteristic unchecked cell growth and metabolic reprogramming of cancer cells. Cancers, including CRC, demonstrate a heightened metabolic dependence, distinguishing them from normal cells [7]. They adopt advanced nutrient acquisition strategies and show increased activity in anabolic pathways. A key aspect of this metabolic reprogramming, observed in almost all cancer types, is the Warburg effect, first identified by Otto Warburg in the 1920s [8,9]. This phenomenon describes the preference of cancer cells for glycolysis over mitochondrial oxidative phosphorylation for ATP production, regardless of oxygen levels [10]. Understanding these metabolic shifts is crucial in addressing the challenges posed by CRC, particularly in the context of metastasis, chemoresistance, and recurrence.

The with-no-lysine (WNK) kinase family in mammals, consisting of serine/threonine kinases WNK1-4, is pivotal in regulating ion homeostasis and blood pressure [11]. Recent studies indicate that malfunctions in WNK can drive the progression of cancer, including tumor growth, metastasis, and angiogenesis, largely through intricate pathways [12]. This includes the phosphorylation of kinase substrates such as SPAK (SPS1-associated proline/alanine-rich kinase) and OSR1 (oxidative stress response kinase 1) [13]. WNK1 significantly impacts the WNT pathway by hindering the degradation of β-catenin, which is essential for cell proliferation, differentiation, and equilibrium maintenance [14]. Its role in ion regulation further drives cancer progression, promoting tumor growth, angiogenesis, T-cell migration, and metastasis [11]. Serum- and glucocorticoid-inducible kinase (SGK1), essential for immune response, metabolic dysregulation, and metastasis, is activated by WNK1 through phosphorylation [14]. In fat cells, Akt3 targets Wnk1 at T58 for phosphorylation, leading to its breakdown. However, without Akt3, WNK1 levels rise, resulting in the phosphorylation of SGK1 and subsequently FOXO1 [15]. This process hinders FOXO1′s nuclear translocation, causing its degradation, which then stimulates fat development and activates peroxisome proliferator–activated receptor gamma (PPARγ) gene transcription [16]. Furthermore, WNK1′s activation of SGK1 induces the expression of Cdc42, a member of the Rho family GTPase, essential in macrophage cell movement through its role in actin formation [17]. Cancer cells exhibit a high demand for glucose, with increased glucose uptake being a hallmark of cancer. The glucose transporter 1 (GLUT1) facilitates glucose absorption in various cell types and is frequently overexpressed in many cancers [18]. In HEK293 cells, WNK1 regulates constitutive glucose uptake by modulating GLUT1 surface expression through its interaction with the Rab GAP TBC1D4 (AS160). WNK1 directly phosphorylates TBC1D4, promoting its association with 14-3-3 regulatory proteins and simultaneously reducing its interaction with the exocytic GTPase Rab8A. This phosphorylation-dependent shift in binding partners relieves TBC1D4-mediated suppression of GLUT1 trafficking to the plasma membrane, thereby increasing GLUT1 surface levels. Importantly, kinase-dead WNK1 mutants fail to induce these effects, demonstrating that WNK1 catalytic activity is essential for TBC1D4-mediated regulation of GLUT1 [19].

The E3 ubiquitin ligase is an enzyme capable of attaching ubiquitin molecules to a lysine residue on a target protein. Typically, this ligase adds several ubiquitin molecules to create polyubiquitin chains on the target protein [20]. These polyubiquitinated proteins are then recognized and broken down by proteasomes [20]. However, in certain instances, the ubiquitin ligases add only a single ubiquitin molecule to a protein, resulting in monoubiquitination [20]. This monoubiquitinated protein is not degraded but may experience changes in its cellular location or function, such as interacting with other proteins that have ubiquitin-binding domains [21]. Seven in absentia homolog 2 (SIAH2) plays a critical role in the ubiquitin-proteasome system, facilitating the ubiquitination of proteins and promoting their degradation. This process influences various cellular processes, including the cell cycle, apoptosis, DNA replication, and signal transduction, and is abnormally activated in various tumor cells [22]. Recently, the SIAH2/WNK1 signaling pathway has been recognized as a crucial modulator of cancer metabolism, especially in terms of glycolysis regulation [23].

This study focuses on elucidating how the functional interplay between SIAH2 and WNK1 modulates glycolytic dependency and survival of drug-resistant CRC stem cells, and whether targeting this axis represents a viable therapeutic strategy. Although SIAH2 and WNK1 have individually been implicated in cancer metabolism and stress adaptation, their coordinated contribution to glycolytic regulation and therapeutic resistance in colorectal cancer stem cells remains insufficiently defined. Addressing this gap is essential for identifying metabolic vulnerabilities that can be therapeutically exploited. The SIAH2/WNK1 pathway has thus emerged as a critical regulator of metabolic adaptation in cancer cells, largely through ubiquitin-dependent signaling and indirect stabilization of hypoxia-inducible factor-1α (HIF-1α), a key mediator of glycolytic gene expression and CSC survival. By dissecting the antagonistic relationship between SIAH2 and WNK1, this study aims to clarify how metabolic regulation contributes to CSC maintenance and drug resistance in CRC. Ultimately, defining the role of the SIAH2/WNK1 axis may facilitate the development of metabolism-targeted therapies designed to overcome chemoresistance and improve clinical outcomes in colorectal cancer.

## 2. Results

### 2.1. SIAH2 and WNK1 Overexpression in CRC Clinical Samples and Cell Lines

**Figure 1:**
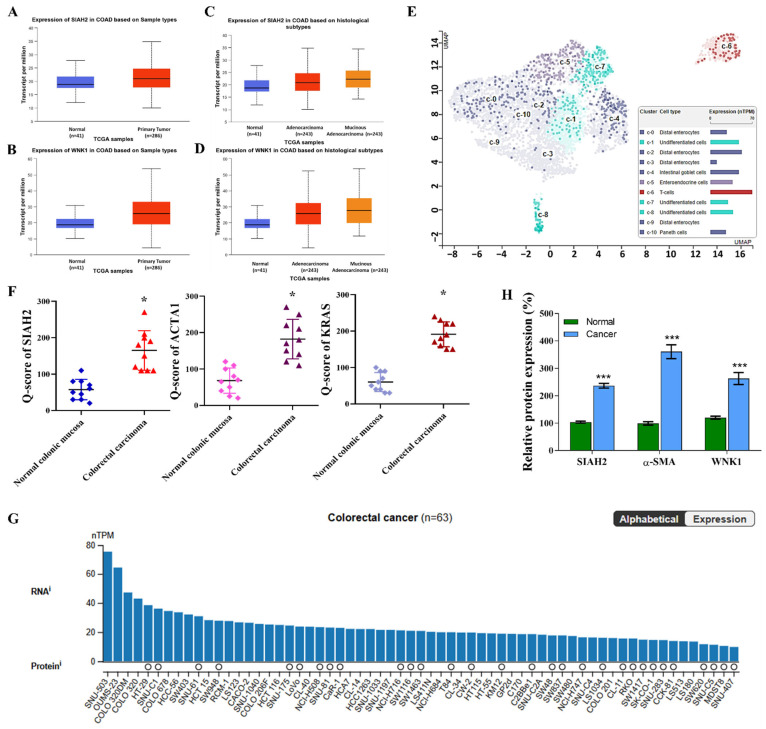
Elevated expression of SIAH2 and WNK1 in colorectal cancer clinical samples and cell line models. (**A**,**B**) Analysis of the TCGA-COAD dataset accessed via UALCAN showing significantly increased *SIAH2* and *WNK1* mRNA expression in primary colorectal adenocarcinoma tissues compared with normal colon tissues. Statistical significance was determined using Student’s *t*-test. (**C**,**D**) Stratification of TCGA-COAD samples by histological subtype demonstrating elevated expression of *SIAH2* and *WNK1* across adenocarcinoma and mucinous adenocarcinoma cases relative to normal tissues. Statistical analysis was performed using one-way ANOVA with appropriate post hoc testing. (**E**) Single-cell RNA-sequencing UMAP illustrating preferential enrichment of *SIAH2* expression in undifferentiated epithelial cell populations, with lower expression in differentiated epithelial lineages. (**F**) Immunohistochemical (IHC) analysis of *SIAH2* protein expression in colorectal cancer tissues, quantified using Q-score assessment, showing significantly higher protein levels in tumor tissues compared with adjacent non-tumor controls. Statistical significance was evaluated using paired Student’s *t*-test. (**G**) Integrated RNA and protein expression analysis of *SIAH2* across colorectal cancer cell lines. Bar plots indicate normalized transcript per million (nTPM) values for individual cell lines, while circles represent protein expression derived from the MS-based Pan-Cancer Atlas dataset; circle size reflects normalized relative protein expression (nRPX), color denotes tissue origin, white circles indicate undetected protein, and absence of a circle indicates unavailable MS data. (**H**) Paired comparison of *SIAH2* mRNA expression between tumor and adjacent non-tumor colorectal tissues from individual patients. Statistical significance was assessed using a paired Student’s *t*-test. Data are presented as mean ± SEM unless otherwise indicated. * *p* < 0.05, *** *p* < 0.001 vs. control.

To investigate the clinical relevance of the seven in absentia homolog 2 (*SIAH2*)–with-no-lysine kinase 1 (*WNK1*) axis in colorectal cancer (CRC), we first analyzed transcriptomic data from The Cancer Genome Atlas–Colorectal Adenocarcinoma (TCGA-COAD) cohort using the UALCAN platform (https://ualcan.path.uab.edu/analysis.html, accessed on 17 August 2025). Bulk RNA-sequencing analysis demonstrated that SIAH2 expression was significantly elevated in primary colorectal tumor tissues compared with matched normal colonic epithelium (**Figure 1A**). This tumor-associated upregulation was consistent across the cohort and reached strong statistical significance. Stratification of TCGA-COAD samples by histological subtype further revealed that *SIAH2* expression remained significantly increased in both adenocarcinoma and mucinous adenocarcinoma relative to normal tissues (**Figure 1B**), indicating that *SIAH2* upregulation is a conserved molecular feature of colorectal malignancy. Parallel analyses of *WNK1* expression revealed a similar pattern. *WNK1* transcripts were significantly higher in tumor tissues than in normal controls (**Figure 1C**), and this elevation persisted across distinct histopathological subtypes (**Figure 1D**). The concordant overexpression of *SIAH2* and *WNK1* at the bulk tumor level supports the premise that these two molecules may operate within a shared regulatory axis in colorectal cancer. To resolve intratumoral heterogeneity and define the cellular compartments contributing to elevated *SIAH2* expression, we next examined single-cell RNA-sequencing datasets. Uniform Manifold Approximation and Projection (UMAP) analysis revealed that *SIAH2* expression was preferentially enriched within undifferentiated epithelial cell clusters, whereas differentiated enterocytes, goblet cells, and Paneth cell populations exhibited comparatively low expression (**Figure 1E**). This spatially resolved analysis suggests that SIAH2 expression is associated with stem-like epithelial subsets within the tumor microenvironment. To validate these findings at the protein level, immunohistochemical staining of colorectal cancer specimens was performed. Semi-quantitative analysis using Q-score assessment demonstrated significantly elevated SIAH2 protein expression in tumor tissues compared with adjacent non-tumor tissues (**Figure 1F**), confirming that SIAH2 upregulation is maintained at the protein level in clinical samples. Integration of RNA and protein expression datasets across different colorectal cancer cells further demonstrated that elevated *SIAH2* transcript levels were generally accompanied by increased protein abundance (**Figure 1G**). Finally, analysis of patient-derived colorectal cancer specimens confirmed widespread SIAH2 expression across clinical samples, with substantial intertumoral variability (**Figure 1H**). This heterogeneity may reflect differences in tumor differentiation status, metabolic dependency, or microenvironmental context.

Collectively, these multi-layered analyses—spanning bulk transcriptomics, histological stratification, single-cell resolution, immunohistochemical validation, and patient specimens—demonstrate that *SIAH2* and *WNK1* are coordinately upregulated in colorectal cancer, with *SIAH2* showing preferential enrichment in undifferentiated, stem-like tumor cell populations. These findings establish a strong clinical and molecular foundation for subsequent functional and mechanistic investigations into the role of the *SIAH2–WNK1* axis in colorectal cancer progression and therapy resistance.

### 2.2. SIAH2 Expression Influences the Aggressive Characteristics of Colorectal Cancer

**Figure 2:**
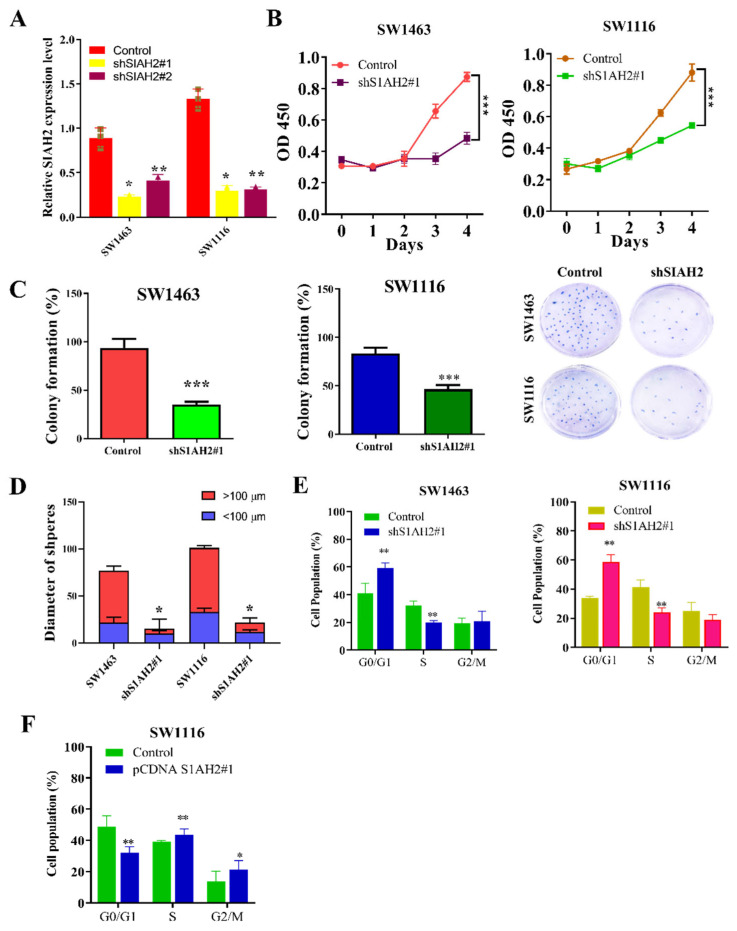
*SIAH2* regulates proliferation, clonogenic growth, tumor sphere formation, and cell-cycle progression in colorectal cancer cells. (**A**) Relative SIAH2 mRNA expression in SW1463 and SW1116 cells after shRNA-mediated knockdown using two independent shRNAs (#1 and #2). (**B**) Cell viability assays showing reduced viability following *SIAH2* silencing. (**C**) Clonogenic assays demonstrating decreased colony-forming ability in *SIAH2*-knockdown cells. (**D**) Tumor sphere formation assays showing reduced sphere number and size after *SIAH2* knockdown. (**E**) Flow cytometric analysis showing G1-phase accumulation and reduced S-phase population following *SIAH2* silencing. (**F**) Flow cytometric analysis showing enhanced cell-cycle progression after *SIAH2* overexpression. Data are presented as mean ± SD (*n* ≥ 3). Statistical significance was determined using Student’s *t*-test or one-way ANOVA with Tukey’s post hoc test. * *p* < 0.05, ** *p* < 0.01, *** *p* < 0.001 vs. control.

Based on the expression profiling presented in **Figure 1**, the colorectal cancer cell lines SW1463 and SW1116, which exhibit relatively high endogenous *SIAH2* expression, were selected for functional studies. To define the biological role of SIAH2, loss-of-function experiments were performed using two independent shRNA constructs targeting *SIAH2* (sh*SIAH2 #1* and sh*SIAH2 #2*), with scrambled shRNA serving as the control. Quantitative RT-PCR analysis confirmed efficient and consistent suppression of *SIAH2* mRNA expression by both shRNA constructs in SW1463 and SW1116 cells (**Figure 2A**), minimizing concerns regarding off-target effects. We next examined the impact of SIAH2 depletion on cell growth. Cell viability assays revealed a significant reduction in viable cell numbers following *SIAH2* knockdown with shRNA2#1 constructs compared with control cells (**Figure 2B**), indicating that *SIAH2* is required for optimal cell survival and proliferation. To assess long-term proliferative capacity, clonogenic assays were performed. *SIAH2*-silenced (shRNA2#1) cells exhibited a marked decrease in colony-forming ability relative to control cells in both CRC cell lines (**Figure 2C**), demonstrating that *SIAH2* is essential for sustained clonogenic growth. Given the close association between clonogenicity and stem-like properties, we further evaluated tumor-initiating potential using three-dimensional tumor sphere formation assays. *shRNA2#1* significantly reduced tumor sphere number and size compared with control cells (**Figure 2D**), indicating impaired self-renewal and sphere-forming capacity upon *SIAH2* depletion. To determine whether the observed growth defects were associated with alterations in cell-cycle progression, flow cytometric analysis was conducted. *SIAH2* knockdown resulted in a significant accumulation of cells in the G1 phase, accompanied by a corresponding reduction in the S-phase population in both SW1463 and SW1116 cells (**Figure 2E**), indicating G1-phase cell-cycle arrest. Conversely, overexpression (OE-) of *SIAH2* led to the opposite phenotype, characterized by a reduced G1-phase population and an increased proportion of S-phase cells, consistent with enhanced cell-cycle progression (**Figure 2F**). Collectively, these findings demonstrate that *SIAH2* positively regulates colorectal cancer cell proliferation, clonogenic survival, tumor sphere formation, and cell-cycle progression, supporting a critical role for *SIAH2* in maintaining aggressive and stem-like cellular phenotypes.

### 2.3. SIAH2 Coordinates WNK1 and Hypoxia-Associated Pathways Relevant to Metabolic Adaptation in Colorectal Cancer

**Figure 3:**
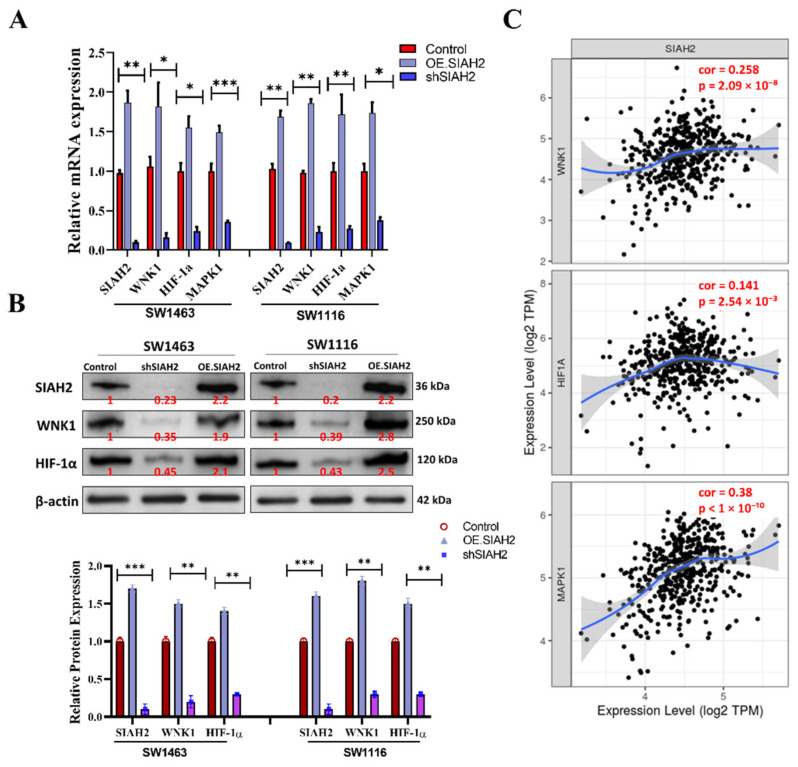
Association of *SIAH2* expression with oncogenic and hypoxia-related transcripts in colorectal cancer. (**A**) Quantitative analysis showing reduced mRNA expression of *WNK1, HIF-1α*, and *MAPK1* following shRNA-mediated knockdown of *SIAH2* in SW1463 and SW1116 cells. (**B**) Representative Western blot images and densitometric quantification demonstrating decreased protein expression of WNK1, HIF-1α, and MAPK1 upon *SIAH2* silencing. Protein levels were normalized to loading controls and expressed relative to control cells. (**C**) Correlation analysis of TCGA-COAD transcriptomic data showing positive associations between *SIAH2* expression and *WNK1* (r = 0.258, r^2^ = 0.067, *p* = 2.09 × 10^−8^), *HIF-1α* (r = 0.141, r^2^ = 0.020, *p* = 2.54 × 10^−3^), and *MAPK1* (r = 0.38, r^2^ = 0.144, *p* < 1 × 10^−10^). Pearson correlation coefficients are shown. All data were obtained from publicly available TCGA-COAD resources, and statistical significance was assessed using Pearson correlation analysis. * *p* < 0.05, ** *p* < 0.01, *** *p* < 0.001 vs. control.

To further elucidate the downstream molecular consequences of *SIAH2* suppression, we examined the effects of shRNA-mediated knockdown of SIAH2 on key signaling molecules associated with oncogenic progression and metabolic adaptation in colorectal cancer cells. In both SW1463 and SW1116 cell lines, silencing of *SIAH2* resulted in a significant reduction in the expression of *WNK1* and hypoxia-associated regulators, including *HIF-1α* and *MAPK1*, as assessed by quantitative transcript analysis (**Figure 3A**). These effects were consistently observed across independent shRNA constructs, supporting the specificity of *SIAH2*-dependent regulation. To validate these findings at the protein level, Western blot analyses were performed. Consistent with the transcriptomic data, *SIAH2* knockdown markedly reduced WNK1, HIF-1α, and MAPK1 protein expression, whereas control cells maintained higher expression levels (**Figure 3B**). Densitometric quantification confirmed reproducible decreases in protein abundance following *SIAH2* depletion, indicating that SIAH2 regulates these signaling components at least in part at the protein level. Given the established roles of WNK1, HIF-1α, and MAPK1 in tumor metabolism, stress adaptation, and proliferative signaling, we next assessed whether the observed regulatory relationships were reflected in clinical datasets. Correlation analyses using TCGA-COAD transcriptomic data revealed significant positive correlations between *SIAH2* expression and *WNK1, HIF-1α,* and *MAPK1* expression levels across patient samples (**Figure 3C**). These associations suggest coordinated activation of *SIAH2*-linked signaling pathways in human colorectal tumors. Collectively, these findings demonstrate that *SIAH2* functions as an upstream modulator of *WNK1*- and hypoxia-associated signaling networks in colorectal cancer cells. The concordance between in vitro loss-of-function experiments and patient-derived transcriptomic correlations supports the existence of a *SIAH2*-centered regulatory axis that integrates oncogenic signaling and adaptive stress responses, providing a mechanistic basis for the metabolic and proliferative phenotypes observed in subsequent sections.

### 2.4. α-SMA-Rich Cancer-Associated Fibroblasts Potentiate SIAH2-Dependent Invasion and Stem-like Properties of Colorectal Cancer Cells

**Figure 4:**
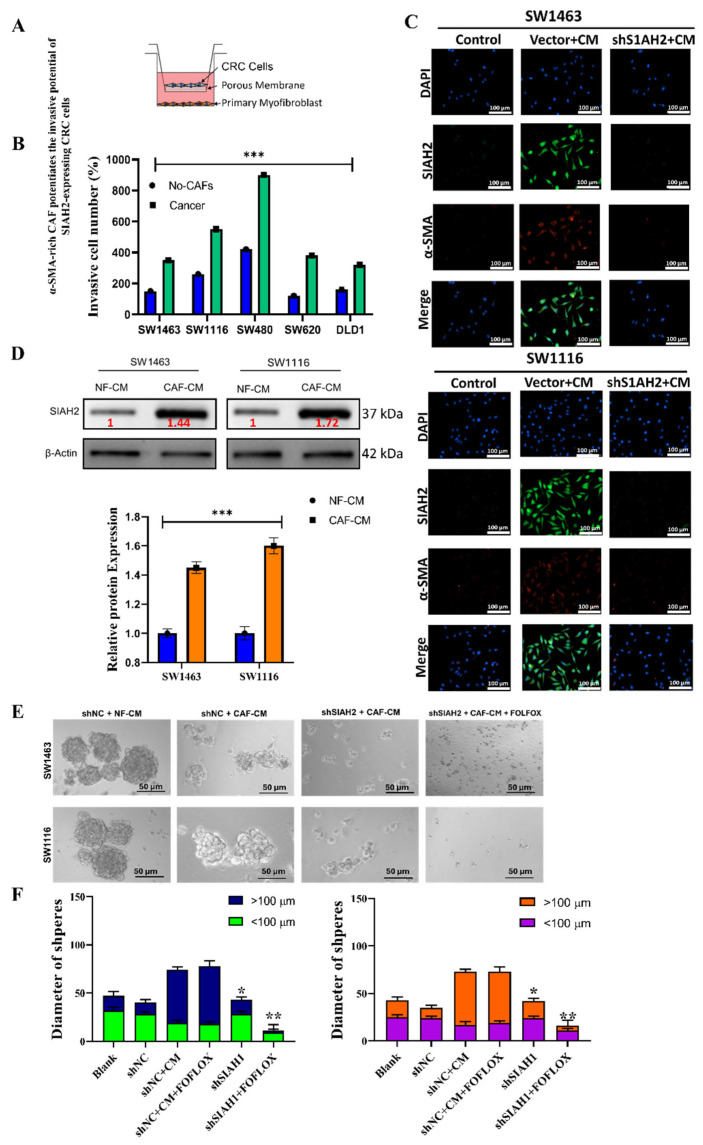
α-SMA-rich CAFs enhance *SIAH2*-dependent invasion and stem-like properties of colorectal cancer cells. (**A**) Schematic of the CRC–CAF Transwell co-culture invasion system. (**B**) CAF co-culture significantly increases invasion of SW1463, SW1116, SW480, SW620, and DLD1 cells (mean ± SEM; ** *p* < 0.001 vs. no-CAF). (**C**) Western blot image of CRC cells under the of CAFs, i.e., NF-CM and CAF-CM. (**D**) Immunofluorescence images showing increased SIAH2 (green) in vector + CM and reduced expression in shSIAH2 + CM groups; nuclei (DAPI, blue), α-SMA (red). Scale bar = 100 µm. (**E**,**F**) Sphere formation assays showing that CAF-conditioned medium promotes large spheroid formation (>100 µm), whereas *SIAH2* knockdown suppresses spheroid growth and increases fluorouracil, leucovorin, and oxaliplatin (FOLFOX) sensitivity (mean ± SEM; * *p* < 0.05, ** *p* < 0.01, *** *p* < 0.001 vs. shNC + CM).

To investigate how the tumor microenvironment modulates *SIAH2*-driven colorectal cancer (CRC) aggressiveness, we examined the impact of α-smooth muscle actin (α-SMA)-positive cancer-associated fibroblasts (CAFs) on the invasive and stem-like behavior of CRC cells. A Transwell co-culture system was established to model paracrine interactions between CRC cells and CAFs (**Figure 4A**). Co-culture of *SIAH2*-expressing CRC cell lines (DLD-1, SW480, HCT116, SNU175, and HT29) with α-SMA-rich CAFs resulted in a significant increase in invasive capacity compared with CRC cells cultured alone or with normal fibroblasts (**Figure 4B**). This enhancement was consistently observed across multiple CRC models, indicating a generalized pro-invasive effect mediated by CAF-derived signals. Immunofluorescence analysis further demonstrated concurrent expression of SIAH2 in CRC cells and α-SMA in adjacent CAFs within the co-culture system (**Figure 4C**), supporting a spatial association between *SIAH2*-positive tumor cells and activated fibroblasts. These findings suggest that CAF-rich microenvironments preferentially support *SIAH2*-expressing CRC cell populations. To further assess CAF-mediated paracrine effects, CRC cells were exposed to conditioned medium (CM) derived from CAFs or normal fibroblasts (NFs). CAF-conditioned medium markedly enhanced migratory and invasive phenotypes, whereas this effect was significantly attenuated upon *SIAH2* knockdown in CRC cells (**Figure 4D**). Notably, reduced *SIAH2* expression also impaired the ability of normal fibroblasts to acquire CAF-like characteristics, suggesting bidirectional signaling between tumor cells and stromal fibroblasts. We next examined whether *SIAH2* contributes to CAF-mediated stem-like phenotypes and therapeutic resistance. Co-culture of SW1463 cells with CAFs and fluorouracil, leucovorin, and oxaliplatin (FOLFOX) resistant CRC cells led to increased *SIAH2* expression and enhanced colon-spheroid formation (**Figure 4E**,**F**). In contrast, *SIAH2* silencing significantly suppressed spheroid formation and increased sensitivity to FOLFOX treatment, indicating that *SIAH2* is required for CAF-driven stemness and chemoresistance. Collectively, these findings demonstrate that α-SMA-positive CAFs potentiate *SIAH2*-dependent invasion, stem-like behavior, and drug resistance in colorectal cancer cells. The reciprocal interaction between *SIAH2*-expressing CRC cells and activated fibroblasts highlights a critical role for the tumor microenvironment–*SIAH2* axis in promoting aggressive CRC phenotypes.

### 2.5. Glycolysis-Related Differential Gene Expression and Protein-Level Validation in Colorectal Cancer

**Figure 5:**
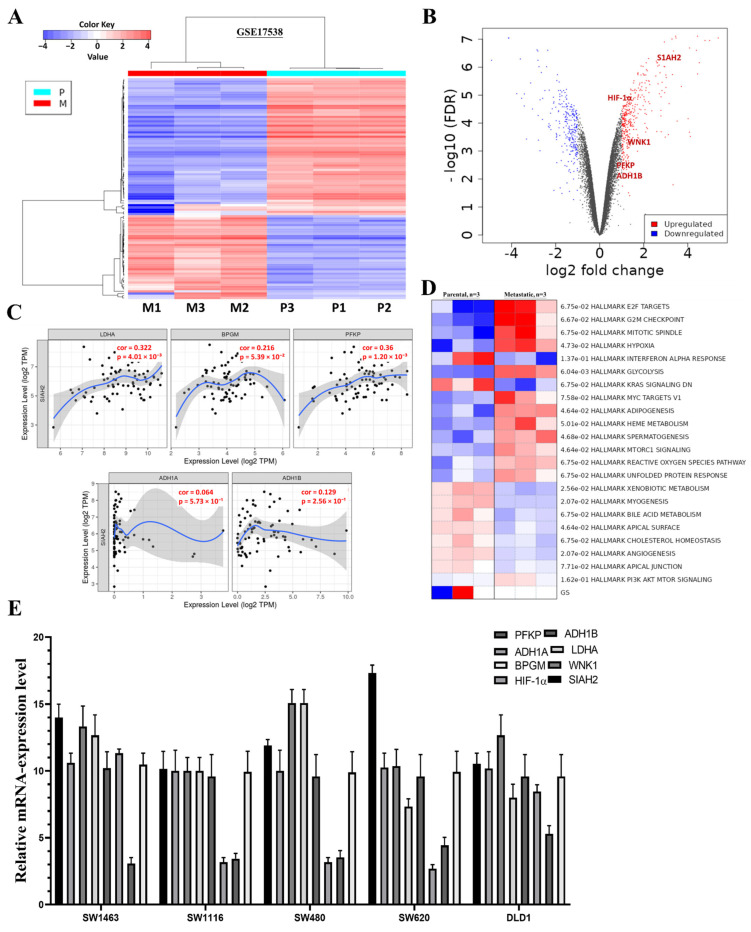
Expression of Glycolysis-Related Genes in Colorectal Cancer. (**A**) Heatmap of differentially expressed genes from GSE17538 comparing parental and metastatic CRC samples (*n* = 3/group). (**B**) Volcano plot highlighting significantly upregulated glycolysis- and hypoxia-related genes (*SIAH2*, *WNK1*, *HIF1A*, *PFKP*, *ADH1B*; FDR < 0.05). (**C**) TCGA-COAD correlation analyses showing positive associations between SIAH2 and glycolytic genes (*PFKP*, *LDHA*, *GAPDH*, *BPGM*, *ADH1A/B*, *HIF1A*). (**D**) GSEA and pathway heatmap demonstrating enrichment of glycolysis and metabolic signaling in metastatic CRC. (**E**) qRT-PCR validation of glycolysis-related genes and *SIAH2/WNK1* in CRC cell lines (mean ± SEM, *n* = 3).

To investigate glycolysis-associated transcriptional alterations in colorectal cancer (CRC), we analyzed the GEO dataset GSE17538, comprising parental and metastatic CRC samples (*n* = 3 per group). Differential expression analysis identified 668 differentially expressed genes (DEGs) with a fold change > 2 and *p* < 0.05, including 397 downregulated and 271 upregulated genes in metastatic samples compared with parental counterparts, as shown in the heatmap and volcano plot (**Figure 5A**,**B**). Among the upregulated DEGs, several key regulators of glycolytic metabolism and hypoxia signaling—*PFKP*, *LDHA*, *GAPDH*, *BPGM*, *ALDH1A/B*, and *HIF1B*—were prominently enriched. Correlation analysis using the TCGA-COAD cohort further demonstrated strong and significant positive associations between these glycolysis-related genes and *SIAH2*, indicating a coordinated metabolic transcriptional program linked to *SIAH2* related signaling (**Figure 5C**). Consistent with these findings, pathway enrichment and gene set enrichment analyses revealed that glycolysis is one of the most significantly upregulated pathways in metastatic CRC samples relative to parental tumors (**Figure 5D**). Heatmap visualization further confirmed increased expression of glycolytic and metabolic pathway signatures in metastatic CRC. These transcriptomic observations were experimentally validated by qRT-PCR analysis in multiple CRC cell lines, which confirmed elevated expression of *SIAH2, WNK1, HIF-1α*, and key glycolytic enzymes (**Figure 5E**). To extend these findings to the protein level, we next examined the impact of SIAH2 modulation on glycolysis-associated protein expression. Western blot analyses demonstrated that shRNA-mediated knockdown of *SIAH2* markedly reduced the protein abundance of glycolytic regulators, whereas *SIAH2* overexpression resulted in a pronounced increase in their expression compared with control cells (**Supplementary Figure S1A**). Densitometric quantification confirmed consistent and reproducible changes in protein levels following both *SIAH2* silencing and overexpression, with stable loading controls across all conditions. Given the important role of the tumor microenvironment in metabolic reprogramming, we further assessed the influence of conditioned medium (CM) on *SIAH2*-dependent metabolic signaling. Under basal conditions without CM (−CM), control cells exhibited relatively low expression of glycolysis-associated proteins. Exposure to CM (+CM) moderately increased protein expression in control cells, indicating microenvironment-driven metabolic modulation (**Supplementary Figure S1B**). Notably, *SIAH2* overexpression in the presence of CM (*SIAH2* + CM) further amplified the expression of glycolytic regulators, whereas *SIAH2* knockdown attenuated CM-induced effects, demonstrating that *SIAH2* is required for maximal microenvironment-mediated activation of glycolytic signaling. Collectively, these transcriptomic, transcriptional, and protein-level analyses establish that *SIAH2–WNK1* signaling is closely associated with enhanced glycolysis-related gene programs in CRC, both intrinsically and in response to microenvironmental cues. These findings provide a mechanistic basis for the functional metabolic alterations assessed in subsequent Seahorse extracellular flux analyses.

### 2.6. SIAH2 Modulation Functionally Regulates Glycolytic Flux and Metabolic Dependency in CRC Cells

**Figure 6:**
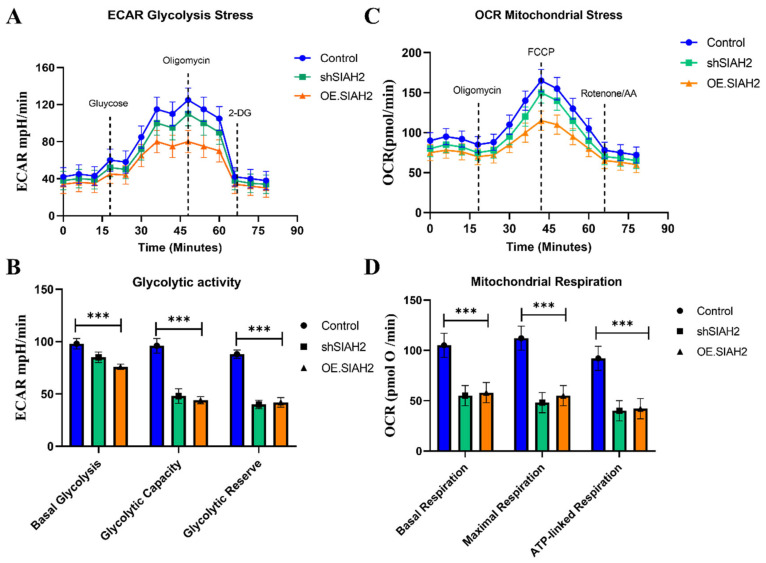
SIAH2 regulates glycolytic metabolism in colorectal cancer cells. (**A**) Seahorse extracellular acidification rate (ECAR) profiles in CRC cells expressing control shRNA, sh*SIAH2*, or OE-*SIAH2* following sequential injections of glucose, oligomycin, and 2-DG. (**B**) Oxygen consumption rate (OCR) profiles measured in parallel. (**C**,**D**) Quantification of glycolytic capacity, glycolytic reserve, and ATP-linked respiration. Data are presented as mean ± SEM from three independent experiments. Statistical analysis was performed using ANOVA with Tukey’s post hoc test. *** *p* < 0.001 vs. control.

Based on the transcriptomic and pathway analyses described in Section 2.5, which identified glycolysis as a key pathway associated with *SIAH2–WNK1* activation in colorectal cancer, we next sought to determine whether direct modulation of *SIAH2* expression functionally alters cellular metabolic behavior. To this end, we performed Seahorse extracellular flux analyses in CRC cells subjected to shRNA-mediated knockdown of *SIAH2* (sh*SIAH2*), *SIAH2* overexpression (*OE-SIAH2*), and corresponding control conditions. Extracellular acidification rate (ECAR) profiling revealed that *SIAH2* knockdown markedly suppressed glycolytic activity, as evidenced by reduced basal ECAR following glucose stimulation and a significant attenuation of glycolytic capacity and glycolytic reserve upon oligomycin treatment (**Figure 6A**). In contrast, *SIAH2* overexpression significantly enhanced ECAR responses, indicating increased glycolytic flux and an expanded glycolytic reserve compared with control cells. These results demonstrate that *SIAH2* positively regulates glycolytic output in CRC cells. Consistent with these findings, oxygen consumption rate (OCR) analyses revealed distinct effects of *SIAH2* modulation on mitochondrial respiration (**Figure 6B**). *SIAH2* knockdown was associated with relatively preserved or modestly increased mitochondrial respiration, whereas *SIAH2* overexpression resulted in reduced ATP-linked respiration and altered maximal respiratory capacity. These changes suggest a metabolic shift toward glycolysis-dominant energy production upon *SIAH2* activation. Quantitative analysis of Seahorse-derived parameters further supported these observations (**Figure 6C**,**D**). Cells overexpressing *SIAH2* exhibited significantly increased glycolytic capacity and reserve, whereas sh*SIAH2* cells displayed diminished glycolytic parameters and greater reliance on mitochondrial respiration. Together, these data indicate that *SIAH2* acts as a key metabolic regulator that biases CRC cells toward glycolytic dependency. Importantly, these functional metabolic alterations are concordant with the glycolysis-related gene expression patterns identified in metastatic CRC samples and TCGA-COAD datasets (Section 2.5), thereby establishing a direct mechanistic link between *SIAH2* expression, glycolytic gene programs, and functional metabolic reprogramming in colorectal cancer cells.

## 3. Discussion

A major finding of this work is the functional integration of *SIAH2* and *WNK1* signaling within the tumor microenvironment (TME), particularly in the context of α-smooth muscle actin (α-SMA)-rich cancer-associated fibroblasts (CAFs). CAFs are increasingly recognized as key drivers of tumor progression, therapy resistance, and poor clinical outcomes across multiple malignancies. Our data demonstrate that α-SMA-positive CAFs actively enhance CRC cell invasion, spheroid formation, and chemoresistance, and that these effects are critically dependent on *SIAH2* activity. These findings extend prior observations linking α-SMA-rich CAFs to aggressive tumor behavior by providing mechanistic evidence that *SIAH2* functions as a molecular mediator connecting stromal activation to cancer cell metabolic and invasive phenotypes. *SIAH2* is a hypoxia-responsive E3 ubiquitin ligase that regulates protein stability in pathways governing cell survival, proliferation, and metabolic adaptation [24]. Our results show that *SIAH2* supports cancer stem–like cell (CSC) traits, including clonogenicity and tumor-sphere formation, while maintaining cell-cycle progression. Mechanistically, *SIAH2* contributes to CSC maintenance by stabilizing *HIF-1α*, thereby enabling cancer cells to survive under hypoxic and nutrient-limited conditions characteristic of the CRC TME [25]. This stabilization promotes glycolytic gene expression and supports the metabolic flexibility required for sustained CSC survival and therapy resistance. Importantly, *SIAH2* depletion reduced these CSC-associated phenotypes, underscoring its functional importance in maintaining the tumor’s regenerative and metastatic capacity.

The coculture and conditioned-medium experiments further reveal that *SIAH2* influences CAF behavior and CAF-mediated signaling, highlighting a bidirectional interaction between cancer cells and the stromal compartment. We observed that *SIAH2* activity is associated with enhanced RAS–ERK pathway signaling, a canonical oncogenic axis known to drive proliferation, invasion, and survival. This observation suggests that *SIAH2* may amplify oncogenic signaling within the TME, thereby reinforcing CAF-driven tumor support and fostering a pro-tumorigenic niche [26]. Elevated expression of *SIAH2* and related signaling components within the tumor stroma aligns with adverse clinical features, supporting the clinical relevance of this axis in CRC progression.

Downstream of *SIAH2* and *WNK1*, our data implicate key mediators of epithelial–mesenchymal transition (EMT) and extracellular matrix remodeling, including Snail (*SNAI1*) and fibronectin (*FN1*), which are markedly upregulated in CRC stroma and correlate with advanced disease and poor survival. Snail is a master regulator of EMT, enabling cancer cells to acquire migratory and invasive capabilities essential for metastasis. Fibronectin, as a major extracellular matrix component, facilitates cell adhesion, migration, and tissue remodeling, further supporting metastatic dissemination. By promoting the expression of these downstream effectors, the *SIAH2/WNK1* axis contributes to both cell-intrinsic invasive programs and TME remodeling, thereby accelerating CRC progression [27]. Targeting this axis may therefore disrupt multiple layers of tumor aggressiveness, from EMT induction to stromal support of metastasis.

Furthermore, the metabolic reprogramming represents another key dimension of *SIAH2/WNK1* function. Consistent with the Warburg effect, CRC cells preferentially rely on aerobic glycolysis to meet their energetic and biosynthetic demands [28]. Our transcriptomic, protein-level, and Seahorse analyses demonstrate that *SIAH2* and *WNK1* cooperatively regulate glycolytic flux, linking hypoxia signaling, ion homeostasis, and metabolic adaptation. *WNK1*, through its roles in ion transport and osmotic stress responses, supports cellular homeostasis under fluctuating TME conditions, while *SIAH2* stabilizes HIF-1α to sustain glycolytic gene expression. Together, these pathways establish a metabolic state that favors tumor growth, CSC maintenance, and resistance to chemotherapy. From a translational perspective, our findings support the concept that dual targeting of *SIAH2* and *WNK1* may provide a more effective therapeutic strategy than targeting either pathway alone. Inhibiting *SIAH2* could destabilize HIF-1α and impair hypoxia-driven glycolysis, while *WNK1* inhibition may further disrupt metabolic and stress-adaptation pathways. Importantly, such a strategy may also attenuate CAF-mediated stromal support, thereby weakening the protective niche that promotes CRC survival and drug resistance. Emerging small-molecule inhibitors targeting *SIAH2* or *WNK1* signaling offer promising starting points for future therapeutic development.

A major strength of this study is the integrated multi-level approach, combining clinical datasets, in vitro functional assays, coculture models, metabolic flux analyses, and bioinformatics to define the role of the *SIAH2/WNK1* axis in CRC. This comprehensive strategy allows us to link molecular mechanisms to functional phenotypes and clinical relevance. However, several limitations should be acknowledged. First, although our in vitro and coculture models capture key aspects of CAF–CRC interactions, in vivo validation in orthotopic or patient-derived xenograft models will be necessary to fully confirm the therapeutic potential of targeting this axis. Second, the patient cohort analyzed for tissue-based validation was relatively limited in size and follow-up duration, which may constrain the interpretation of clinical correlations. Finally, while our data establish a strong association between *SIAH2/WNK1* signaling and metabolic reprogramming, future studies using stable isotope tracing and in vivo metabolic profiling will further refine our understanding of pathway-specific metabolic dependencies.

**Figure 7:**
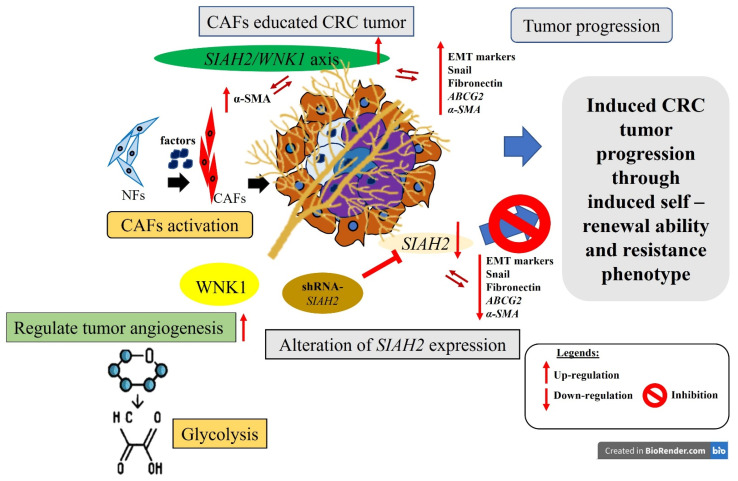
Study Schematic Overview. This study identifies the SIAH2/WNK1 gene pair as a potential prognostic biomarker and regulatory target in colorectal cancer (CRC). Targeting both genes concurrently, rather than individually, could substantially hinder CRC progression, metastasis, and recurrence. This effect is mediated by modulating the expression of epithelial–mesenchymal transition (EMT) markers and cancer-associated fibroblasts (CAFs) within the CRC tumor microenvironment (TME). Created in BioRender. Yadav, D. V. K. (2026) https://BioRender.com/tp6iy1y.

In summary, our findings, as schematically illustrated in **Figure 7**, identify the *SIAH2–WNK1* signaling axis as a central regulator of colorectal cancer progression by integrating metabolic reprogramming, cancer stem cell maintenance, epithelial–mesenchymal transition (EMT), and tumor microenvironment (TME) remodeling. We demonstrate that aberrant activation of *SIAH2* and *WNK1* reinforces glycolytic dependency and hypoxia adaptation, sustains stem-like tumor cell populations, and amplifies CAF-driven stromal support, collectively promoting invasion, therapeutic resistance, and disease recurrence. Importantly, simultaneous targeting of *SIAH2* and *WNK1* emerges as a promising therapeutic strategy capable of disrupting both tumor-intrinsic survival mechanisms and the supportive stromal niche. By impairing CSC maintenance, attenuating EMT-associated invasiveness, and weakening CAF-mediated signaling within the TME, this dual-target approach has the potential to produce more durable antitumor responses than strategies focused on cancer cells alone. Together, the mechanistic and functional evidence presented in this study provides a strong rationale for further preclinical and clinical evaluation of *SIAH2/WNK1* co-targeting as an innovative treatment paradigm for colorectal cancer.

## 4. Materials and Method

### 4.1. Bioinformatics Analysis

Differential gene expression and clinical correlation analyses were performed using publicly available colorectal cancer datasets. Bulk RNA-sequencing data from TCGA–Colorectal Adenocarcinoma (TCGA-COAD) were accessed through the UALCAN portal (https://ualcan.path.uab.edu/analysis.html, accessed on 17 August 2025) [29] to evaluate the expression of *SIAH2*, *WNK1*, and glycolysis-associated genes across normal and tumor tissues, as well as across histological subtypes. For metastatic-associated transcriptional profiling, the Gene Expression Omnibus (GEO) dataset GSE17538 was downloaded from the National Center for Biotechnology Information (NCBI) (Bethesda, MD, USA; https://www.ncbi.nlm.nih.gov/geo/, accessed on 17 August 2025), comprising parental and metastatic CRC samples (*n* = 3 per group). Differential expression analysis was conducted using R software (version 4.2.2; R Foundation for Statistical Computing, Vienna, Austria) with limma (version 3.54.2) using thresholds of |log_2_ fold change| > 1 and *p* < 0.05. Heatmaps and volcano plots were generated using ggplot2 (version 3.4.0) and pheatmap (version 1.0.12) packages in R. Correlation analyses between SIAH2 and glycolysis-related genes (*PFKP*, *LDHA*, *GAPDH*, *BPGM*, *ADH1A/B*, and *HIF1A*) were performed using TIMER2.0 (https://cistrome.shinyapps.io/timer/, Dana-Farber Cancer Institute, Boston, MA, USA; accessed on 17 August 2025). Gene set enrichment analysis (GSEA) was conducted using GSEA software (version 4.3.2; Broad Institute, Cambridge, MA, USA) with MSigDB Hallmark gene sets (version 2023.1), with a false discovery rate (FDR) < 0.05 considered statistically significant. Single-cell RNA-sequencing expression patterns were visualized using publicly available CRC scRNA-seq datasets curated through UALCAN and annotated using CellMarker (http://bio-bigdata.hrbmu.edu.cn/CellMarker/, Harbin Medical University, Harbin, China; accessed on 17 August 2025).

### 4.2. Patients and Samples

Patients who underwent primary curative surgical resection for colorectal cancer (CRC) at the Department of Surgery, Taipei Medical University–Shuang Ho Hospital were prospectively enrolled. Exclusion criteria included non-curative resections (R1/R2), inadequate lymph node dissection (<12 retrieved nodes), a history of ulcerative colitis, concurrent malignancies, receipt of neoadjuvant therapy, or perioperative mortality defined as hospital stay <30 days before death. Only patients with pathologically confirmed colon adenocarcinoma who received curative-intent surgery were included in the analysis. Paired tumor tissues and adjacent non-tumor colonic mucosa were collected intraoperatively. Tumor recurrence was monitored by routine clinical imaging, including computed tomography, radiography, and ultrasonography, according to institutional follow-up protocols. All patients were followed for survival outcomes, with a median follow-up duration of 16.9 months (range, 2.7–36.7 months). This study was conducted in accordance with institutional ethical standards and the Declaration of Helsinki and was approved by the TMU Joint Institutional Review Board (TMU-JIRB) (Approval Code: 201301046, Approval Date: 28 June 2013 & Approval Code: 201503047, Approval Date: 21 May 2015). Written informed consent was obtained from all participants prior to sample collection. For tissue-based gene expression analyses, tumor and matched non-tumor mucosa were immediately preserved following surgical resection and processed for total RNA extraction, followed by quantitative reverse transcription PCR (qRT-PCR). For protein-level analyses, tissue lysates were prepared from paired samples under standardized lysis conditions. Protein abundance was quantified after normalization to total protein input, and expression levels were reported as relative protein expression between tumor and corresponding non-tumor tissues.

### 4.3. Cell Culture

Human CRC cell lines HT-29 (ATCC HTB-38™, Cellosaurus CVCL_0320), HCT-116 (ATCC CCL-247™, CVCL_0291), SW480 (ATCC CCL-228™, CVCL_0546), SW620 (ATCC CCL-227™, CVCL_0547), DLD-1 (ATCC CCL-221™, CVCL_0248), and SNU-175 (ATCC CRL-2236™, CVCL_5031) were purchased from the American Type Culture Collection (ATCC, Manassas, VA, USA). The corresponding genetic and authentication information for these lines is available in the Cellosaurus database (Database name: Cellosaurus; accession numbers: CVCL_0320, CVCL_0291, CVCL_0546, CVCL_0547, CVCL_0248, and CVCL_5031). Cells were cultured in RPMI-1640 medium (Gibco™, Thermo Fisher Scientific, Waltham, MA, USA) supplemented with 10% fetal bovine serum (FBS; Gibco™, Thermo Fisher Scientific), 100 U/mL penicillin, 100 μg/mL streptomycin, and 20 mM L-glutamine (all from Thermo Fisher Scientific, Waltham, MA, USA). Cells were maintained at 37 °C in a humidified incubator with 5% CO_2_.

### 4.4. Cell Viability Assessment Using Cell Counting Kit-8

To evaluate cell survival and growth in our colorectal cancer (CRC) research, we employed the Cell Counting Kit-8 (CCK-8) assay from Dojindo Laboratories, Kumamoto, Japan. Known for its high sensitivity and non-radioactive properties, this assay is widely used in cell biology for accurately and effectively measuring cell viability. CRC cells (2–5 × 10^3^ cells/well) were seeded in 96-well plates, and absorbance at 450 nm using a SpectraMax microplate reader (Molecular Devices, San Jose, CA, USA). Data were normalized to control groups.

### 4.5. Glycolytic Flux and Seahorse Extracellular Flux Analysis

Cellular glycolytic and mitochondrial functions were evaluated using the Seahorse XF extracellular flux analyzer following the manufacturer’s protocols (Agilent Technologies, Santa Clara, CA, USA). Data acquisition and analysis were performed using Wave software (version 2.6.1, Agilent Technologies, Santa Clara, CA, USA). CRC cells were seeded into Seahorse XF assay plates at optimized densities and cultured overnight. Prior to analysis, cells were washed and incubated in Seahorse assay medium supplemented with appropriate substrates and equilibrated in a non-CO_2_ incubator for 45–60 min. For assessment of glycolytic function, cells were subjected to the Glycolysis Stress Test, involving sequential injections of glucose to initiate glycolysis, oligomycin to inhibit mitochondrial ATP synthase and drive maximal glycolytic flux, and 2-deoxyglucose (2-DG) to inhibit hexokinase and terminate glycolysis. The extracellular acidification rate (ECAR) was continuously recorded and used to calculate parameters including basal glycolysis, glycolytic capacity, and glycolytic reserve. Mitochondrial respiration was assessed using the Mito Stress Test, with sequential injections of oligomycin, FCCP, and rotenone/antimycin A to determine basal respiration, ATP-linked respiration, maximal respiratory capacity, and spare respiratory capacity based on changes in the oxygen consumption rate (OCR). Following Seahorse analysis, cells were lysed and total protein content was quantified using a BCA assay. ECAR and OCR values were normalized to protein content to account for differences in cell number between wells. Each experimental condition was analyzed in multiple technical replicates and repeated in at least three independent biological experiments.

### 4.6. Co-Culture of CRC Cells with CAFs

CRC–fibroblast co-culture experiments were performed by adapting the method described by Sung et al. [30]. Briefly, cancer-associated fibroblasts (CAFs) were seeded at a density of 8 × 10^4^ cells in 10 cm culture dishes and allowed to adhere for 24 h. CRC cells were then added at a density of 4 × 10^3^ cells per dish, with the CAF-to-cancer cell ratio adjusted according to cell type (ranging from 50:1 to 10:1). Following co-culture initiation, the medium was refreshed to remove non-adherent cells, and cultures were maintained under standard conditions. For conditioned medium (CM) experiments, CAFs were cultured to indicated confluency, washed with phosphate-buffered saline, and incubated in fresh complete medium for a defined conditioning period. The resulting supernatants (CAF-CM) were collected, clarified by centrifugation to remove cellular debris, and either used immediately or stored at −80 °C to preserve bioactivity. Normal fibroblast–conditioned medium (NF-CM) was prepared in parallel using identical procedures. CRC cells were exposed to CAF-CM or NF-CM for the durations specified in each assay prior to downstream functional analyses.

### 4.7. Short Hairpin (sh-) RNA Interference and Transfection of Overexpression Plasmids

Stable knockdown of *SIAH2* was achieved using commercially available lentiviral short hairpin RNA (shRNA) constructs targeting human SIAH2. Human SIAH-2 shRNA plasmids (sc-37497-SH) and SIAH-2 shRNA lentiviral particles (sc-37497-V), together with a matched non-targeting scrambled shRNA control, were obtained from Santa Cruz Biotechnology (Dallas, TX, USA). These reagents contain multiple shRNA sequences targeting distinct regions of the *SIAH2* transcript. Two independent SIAH2-targeting shRNA clones (sh*SIAH2* #1 and sh*SIAH2* #2) were used in all experiments to minimize off-target effects and ensure knockdown specificity. CRC cells were transduced with *SIAH2* shRNA lentiviral particles in the presence of polybrene (8 µg/mL; Sigma-Aldrich, St. Louis, MO, USA) to enhance infection efficiency. After 48–72 h, transduced cells were selected using puromycin (Thermo Fisher Scientific, Waltham, MA, USA) at concentrations optimized for each cell line. Stable knockdown efficiency was confirmed at both the mRNA and protein levels by quantitative RT-PCR and Western blot analysis, respectively. For gain-of-function experiments, the full-length human *SIAH2* cDNA was cloned into the mammalian expression vector pcDNA™3.1 (+) (Invitrogen™, Thermo Fisher Scientific; Cat. No. V79020), which drives constitutive gene expression under the CMV promoter. Cells transfected with empty pcDNA™3.1 (+) vector served as vector controls. Transient transfection was performed using Lipofectamine™ 2000 transfection reagent (Invitrogen™, Thermo Fisher Scientific; Cat. No. 11668-027) according to the manufacturer’s instructions. Briefly, plasmid DNA–lipid complexes were prepared in Opti-MEM^®^ Reduced Serum Medium (Thermo Fisher Scientific) and added to cells at 70–80% confluency. Cells were harvested 24–48 h post-transfection for validation of *SIAH2* overexpression by qRT-PCR and immunoblotting. Cells with stable *SIAH2* knockdown or transient *SIAH2* overexpression were subsequently used for downstream functional assays, including cell proliferation, invasion, clonogenic survival, tumor sphere formation, Seahorse metabolic flux analysis, and chemotherapeutic response assays.

### 4.8. RNA Isolation and Reverse Transcription Quantitative Polymerase Chain Reaction (RT–qPCR)

Total RNA was extracted from cultured colorectal cancer cells using TRIzol™ reagent (Life Technologies, Thermo Fisher Scientific, Waltham, MA, USA) according to the manufacturer’s instructions. RNA concentration and purity were assessed by spectrophotometric measurement of absorbance at 260 and 280 nm, and only samples with A260/A280 ratios between 1.8 and 2.0 were used for subsequent analyses. For complementary DNA (cDNA) synthesis, 200 ng of total RNA was reverse-transcribed using the OneStep RT-PCR Kit (QIAGEN, Hilden, Germany; Taiwan branch) following the manufacturer’s protocol. Quantitative real-time PCR was performed using the Rotor-Gene SYBR^®^ Green PCR Kit (QIAGEN) on a Rotor-Gene Q real-time PCR system (QIAGEN). Each reaction was carried out in a final volume recommended by the manufacturer and included gene-specific forward and reverse primers. All qPCR reactions were performed in technical triplicates, and at least three independent biological replicates were analyzed for each experimental condition. Melting curve analysis was conducted at the end of each amplification run to verify primer specificity and the absence of nonspecific amplification or primer–dimer formation. Gene expression levels were normalized to GAPDH as the internal reference gene. Relative mRNA expression was calculated using the 2^−ΔΔCt ^ method and expressed as fold change relative to the corresponding control samples. Gene-specific primers were designed to span exon–exon junctions when possible to avoid genomic DNA amplification. The primer sequences, 5′-TTTCCCTGTAAGTATGCCACCAC (forward) and 5′-GTTCCCATTCAACTCCAGTCTG (reverse) for *SIAH2*, 5′-CGTCTGGAACACTTAAAACGTATCT (forward) and 5′-CACCAGCTTCTTAGAACTTTGATCT (reverse) for *WNK1*; 5′-GTTGGTGCTGTTGGCATGGC (forward), 5′-GTGATAATGACCAGCTTGGAG (reverse) for LDHA; 5′-GGACGCGGACGACTCCCGGGC (forward) and 5′-GTCAGACACTCCAGGGCTGCACATGTTCC (reverse) for *PFKP*; 5′-ATCAGAAACTCAACAGCGAAGG (forward) and 5′-TGTGAATGGACCGATTAAGGAC (reverse) for *BPGM* 5′; 5′-AGTCACGCGTGGAGCTAGGTATAGTTGATG (forward) and 5′-AGTCCTCGAGTCCTTGTGGATTTCTTCC (reverse) for *ADH1A*; 5′-CATCAACCCTCAAGACTACAAGAA (forward) and 5′-GCGTCCAGTCAGTAGCAGCATAG (reverse) for *ADH1B*; 5′-CATAAAGTCTGCAACATGGAAGGT (forward) and, 5′-ATTTGATGGGTGAGGAATGGGTT (reverse) for *HIF-1α*; 5′-GTGGGAGTGGGTGGAGGC (forward) and reverse, 5′-TCAACTGGTCTCAAGTCAGTG (reverse) for β-actin and 5′-GGTCTCCTCTGACTTCAACA (forward) and 5′-AGCCAAATTCGTTGTCATAC (reverse) for *GAPDH*.

### 4.9. Western Blot Analysis

Total cellular proteins were extracted using RIPA lysis buffer supplemented with protease inhibitors, clarified by centrifugation, and quantified to ensure equal protein loading. Equal amounts of protein were resolved by SDS–PAGE, transferred onto PVDF membranes, and subjected to immunoblotting. Membranes were probed with primary antibodies against *SIAH2*, *WNK1*, *HIF-1α*, *LDHA*, *PFKP*, cleaved *PARP*, cleaved caspase-3, and *β-actin* (loading control), followed by incubation with HRP-conjugated secondary antibodies. Immunoreactive bands were visualized using enhanced chemiluminescence (ECL substrate, Thermo Fisher Scientific, Waltham, MA, USA) and imaged under non-saturating conditions. Primary antibodies and HRP-conjugated anti-rabbit and anti-mouse IgG secondary antibodies together with the dilution used and molecular weight were listed in **Supplementary Table S1**. Band intensities were quantified by densitometric analysis, normalized to β-actin, and expressed relative to the corresponding control group. For experiments involving CAF-conditioned medium, CRC cells were cultured under −CM or +CM conditions prior to protein extraction to evaluate stromal-induced alterations in signaling pathways.

### 4.10. Cell Invasion, Migration, and Colony Formation Assays

Cell invasion was assessed using a Matrigel-coated Boyden chamber assay, following the method originally described by Albini et al., with minor adaptations. CRC cells subjected to the indicated genetic manipulations were seeded into the upper chambers, while chemoattractant medium—with or without cancer-associated fibroblasts (CAFs)—was placed in the lower chambers. After incubation, invaded cells on the lower surface of the membrane were fixed, stained, and quantified. For colony formation assays, CRC cells (500 cells per well) expressing control vectors, sh*SIAH2*, or *SIAH2* overexpression constructs were seeded into six-well plates and cultured for 7–10 days. Colonies were fixed with paraformaldehyde and stained, and only colonies exceeding a predefined size threshold were counted. Colony numbers were normalized to corresponding control groups and expressed as percentage colony formation. Representative images were captured using identical acquisition settings prior to quantification.

### 4.11. Cell-Cycle Analysis by Flow Cytometry

After stable transfection with shRNA or overexpression constructs, along with control (vector-only) CRC cells, the cells were cultured in six-well plates with RPMI-1640 medium containing 10% fetal bovine serum and incubated at 37 °C in a 5% CO_2_ atmosphere for 24 h. Apoptosis was assessed using the PE Annexin V Apoptosis Detection Kit I (BD Biosciences) according to the manufacturer’s instructions. Cells were detached with 0.25% trypsin-EDTA, washed twice in cold phosphate-buffered saline, and stained with 5 μL Annexin V-PE and 5 μL 7-AAD in binding buffer. After a 15 min incubation at room temperature, apoptosis was analyzed using a BD FACS Aria III flow cytometer. Further, CRC cells subjected to control, shSIAH2 knockdown, or SIAH2 overexpression were harvested, washed with cold PBS, and fixed in cold ethanol. Fixed cells were treated with RNase and stained with propidium iodide (PI) for DNA-content analysis. Flow-cytometric acquisition was performed using linear fluorescence detection, and cell-cycle distributions (G0/G1, S, G2/M) were calculated using standardized DNA-content modeling with identical gating parameters across all samples.

### 4.12. Spheroid Formation and Quantification

To evaluate cancer stem cell-associated self-renewal, CRC cells were cultured under sphere-forming conditions. Experimental groups included shNC + NF-CM, shNC + CAF-CM, sh*SIAH2* + CAF-CM, and sh*SIAH2* + CAF-CM + FOLFOX. Phase-contrast images were acquired at matched time points using identical magnification and imaging parameters. Spheroids were quantified based on number and size, using predefined diameter thresholds (<100 µm and >100 µm). Multiple fields per well and replicate wells per condition were analyzed. Data are presented as mean values derived from independent biological replicates.

### 4.13. Chemotherapy Treatment Under CAF-Conditioned Medium

To assess CAF-mediated chemoresistance, CRC cells cultured in CAF-conditioned medium were treated with the FOLFOX regimen using the same grouping strategy applied to spheroid assays. Treatment timing and dosing were standardized across conditions. Following treatment, spheroid formation and size distribution were quantified using identical imaging and scoring criteria, enabling direct comparison of chemotherapy responsiveness under CAF-derived stimulation.

### 4.14. Immunofluorescence Analysis

CRC cells were seeded onto chamber slides and cultured for 24 h prior to fixation with 2% paraformaldehyde. Cells were permeabilized, blocked, and incubated with primary antibodies against *SIAH2* and *α-SMA*, followed by fluorophore-conjugated secondary antibodies. Nuclei were counterstained with DAPI. For conditioned-medium experiments, cells were treated as indicated (Control, Vector + CM, sh*SIAH2* + CM). Fluorescence images were captured using a Zeiss AxioPhot fluorescence microscope (Carl Zeiss, Oberkochen, Germany). Image acquisition and processing were conducted using ZEN software (version 3.4, Carl Zeiss, Oberkochen, Germany), under identical exposure and gain settings across experimental groups. Channel merging was performed to assess expression patterns and spatial relationships.

### 4.15. Statistical Analysis

Statistical analyses were performed using GraphPad Prism (v8.4.2; GraphPad Software, San Diego, CA, USA) and RStudio (version 2025.09.0+387; Posit Software, Boston, MA, USA). Data are presented as mean ± SD from at least three independent biological replicates unless otherwise stated. Comparisons between two groups were performed using paired or unpaired two-tailed Student’s t-tests, as appropriate. Multi-group comparisons were analyzed using one-way or two-way ANOVA, followed by Tukey’s post hoc test. For analyses involving multiple testing—such as gene-expression correlations, transcriptomic profiling, and pathway enrichment—false discovery rate (FDR) correction was applied using the Benjamini–Hochberg method. Pearson correlation analyses were conducted using log_2_-transformed expression values. Adjusted p values (q < 0.05) were considered statistically significant. Exact statistical tests, sample sizes, and significance thresholds are detailed in the corresponding figure legends.

## 5. Conclusions

This study identifies the SIAH2–WNK1 axis as a key driver of glycolytic reprogramming and cancer stem–like phenotypes in colorectal cancer, integrating hypoxia signaling with tumor–stroma crosstalk. We demonstrate that SIAH2 enhances glycolytic flux, supports proliferation and spheroid formation, and promotes resistance to fluorouracil, leucovorin, and oxaliplatin (FOLFOX), particularly under α-smooth muscle actin (α-SMA)-positive cancer-associated fibroblast (CAF) stimulation. Collectively, these findings highlight SIAH2–WNK1 as a mechanistically linked and therapeutically actionable pathway that may be targeted to impair metabolic dependency and overcome microenvironment-driven chemoresistance in colorectal cancer.

## Data Availability

The datasets used and analyzed in the current study are publicly accessible as indicated in the manuscript. All-experimental procedures, supporting datasets, and additional methodological details are provided in the Supplementary Materials. All authors have reviewed and approved the final version of the manuscript and take collective responsibility for the accuracy, integrity, and reproducibility of the data presented in this study.

## Supplementary Materials

The following supporting information can be downloaded at: https://www.mdpi.com/article/10.3390/ijms27021065/s1.

## Abbreviations

Colorectal cancer (CRC); Cancer stem cells (CSCs); Cancer Genome Atlas Colon Adenocarcinoma (TCGA-COAD); cancer-associated fibroblast (CAF); extracellular matrix (ECM); epithelial–mesenchymal transition (EMT); M2 polarization of macrophages (M2 TAMs); institutional review board (IRB); hypoxia-inducible factor-1 alpha (HIF-1α); α-smooth muscle actin (α-SMA); tumor microenvironment (TME).
