# Supplementary material for: SIAH2–WNK1 Signaling Drives Glycolytic Metabolism and Therapeutic Resistance in Colorectal Cancer
Source: Int J Mol Sci. 2026 Jan 21;27(2):1065. doi: 10.3390/ijms27021065 (PMC12842563; doi:10.3390/ijms27021065)
Supplement: Supplementary file 1 [file ijms-27-01065-s001.zip › ijms-4060761-supplementary.pdf]

Supplementary Information

SIAH2–WNK1 Signaling Drives Glycolytic Metabolism and Therapeutic Resistance in Colorectal Cancer

Kee-Thai Kiu [1,2,†], Cheng-Ying Chu [3,4,†], Yi-Chiao Cheng [5], Min-Hsuan Yen [1,2], Ying-Wei Chen [1,2], Narpati Wesa Pikatan [6], Vijesh Kumar Yadav [1,2], Tung-Cheng Chang [1,2,*]

[1] Division of Colorectal Surgery, Department of Surgery, Taipei Medical University Shuang-Ho Hospital, New Taipei City 235, Taiwan; kiubabar@gmail.com (K.-T.K.); 17251@s.tmu.edu.tw (M.-H.Y.); 16402@s.tmu.edu.tw (Y.-W.C.); vijeshp2@gmail.com (V.K.Y.)

[2] Division of General Surgery, Department of Surgery, School of Medicine, College of Medicine, Taipei Medical University, Taipei 110, Taiwan

[3] CRISPR Gene Targeting Core, Taipei Medical University, Taipei 110, Taiwan; cchu@tmu.edu.tw

[4] TMU Research Center of Cancer Translational Medicine, Taipei Medical University, Taipei 110, Taiwan

[5] Division of Colon and Rectal Surgery, Department of Surgery, Tri-Service General Hospital, National Defense Medical University, Taipei 114, Taiwan; ndmcjoe@gmail.com

[6] Division of Urology, Department of Surgery, Faculty of Medicine, Universitas Gadjah Mada, Yogyakarta 55281, Indonesia; narpatiwp@gmail.com

* Correspondence: rousse11@tmu.edu.tw; Tel.: +886-2-2490088 (ext. 2919); Fax: +886-2-2248-0900

† These authors contributed equally to this work.

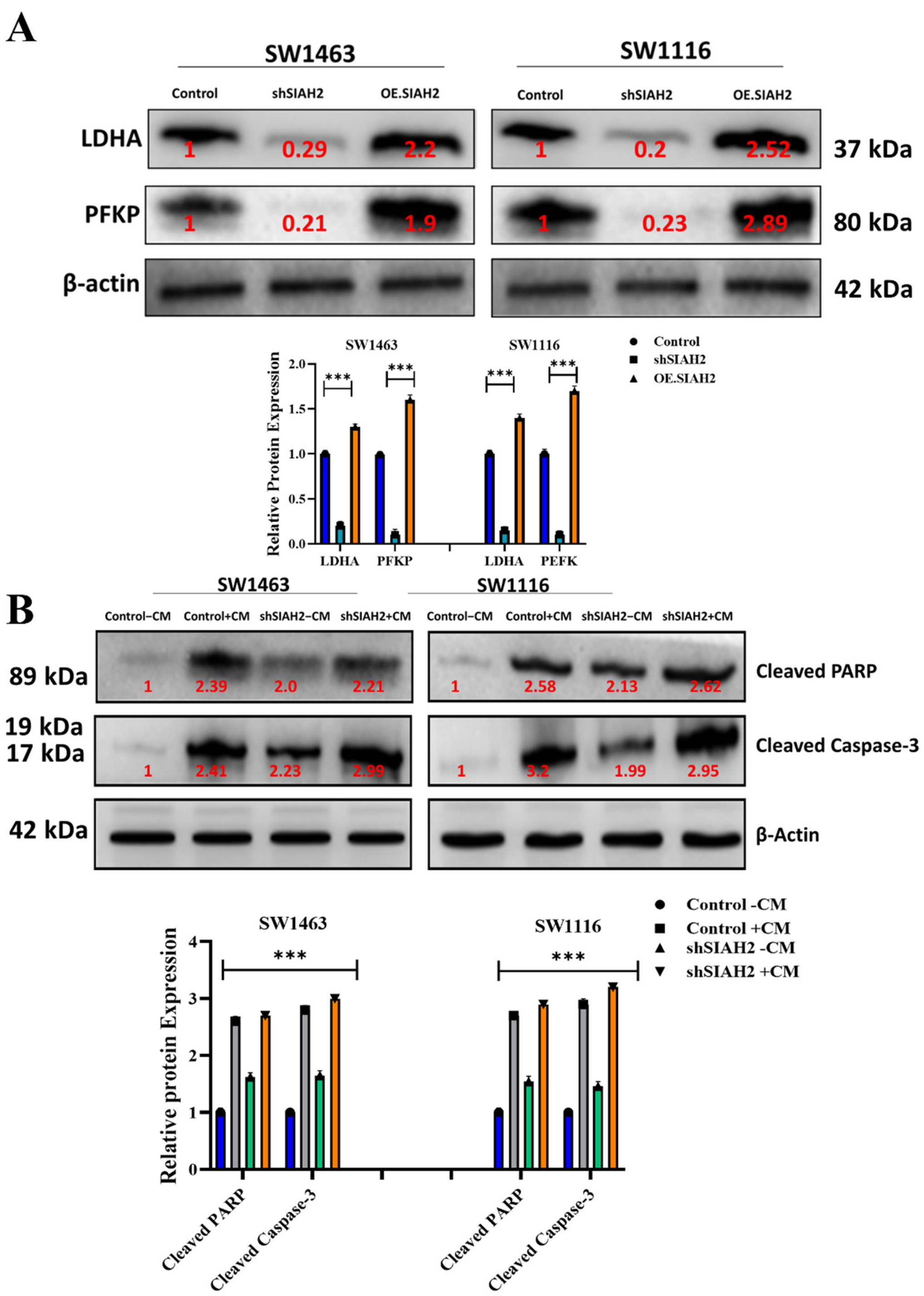

Supplementary Figure S1. SIAH2 modulation regulates glycolysis-associated protein expression in CRC cells. (A) Representative Western blot images showing the effects of shRNA-mediated SIAH2 knockdown (sh#1, sh#2) and SIAH2 overexpression (OE) on glycolysis-related protein expression in colorectal cancer cells. β-Actin was used as a loading control. Red numbers indicate relative densitometric values normalized to control. (B) Effects of conditioned medium (CM) on SIAH2-dependent glycolytic protein expression. CRC cells were cultured under −CM or +CM conditions following SIAH2 knockdown or overexpression. Quantitative densitometric analysis is shown as fold change relative to control. Data represent mean ± SEM from at least three independent experiments. Statistical significance was determined using Student's t-test or one-way ANOVA (p < 0.05, p < 0.01, *p < 0.001).

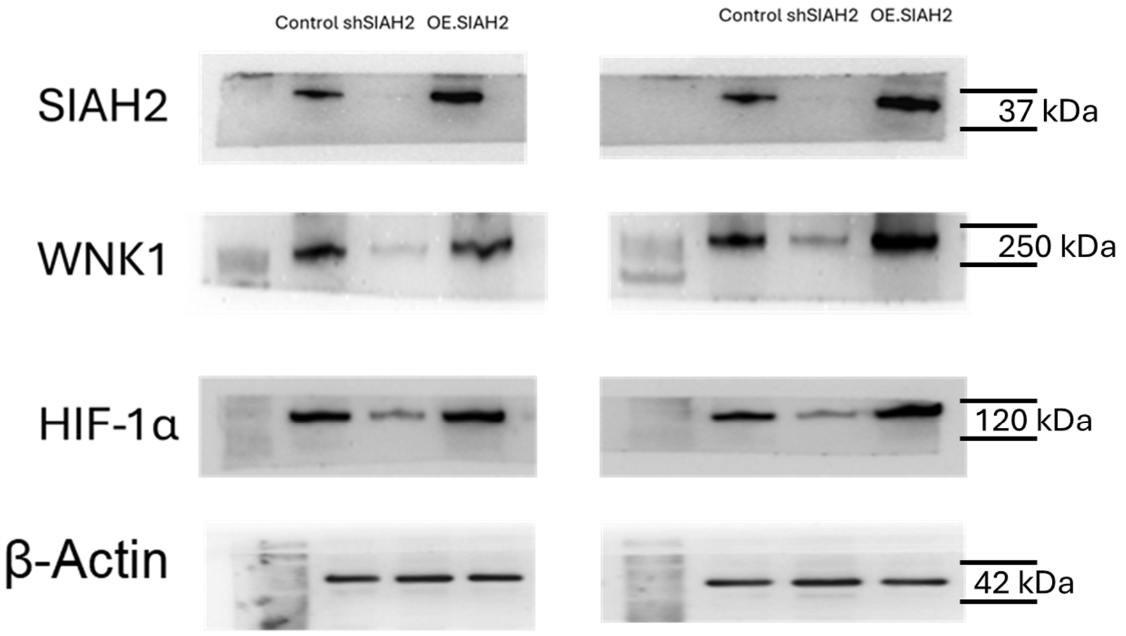

Supplementary Figure S2: Raw uncropped western blot image for supplementary **Figure 3B**.

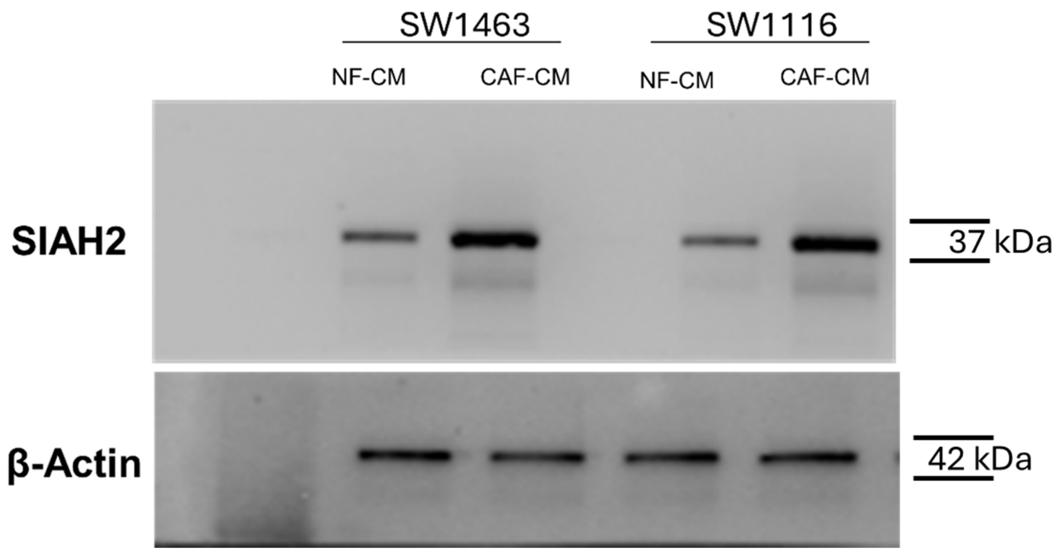

Supplementary Figure S3: Raw uncropped western blot image for supplementary **Figure S4C**.

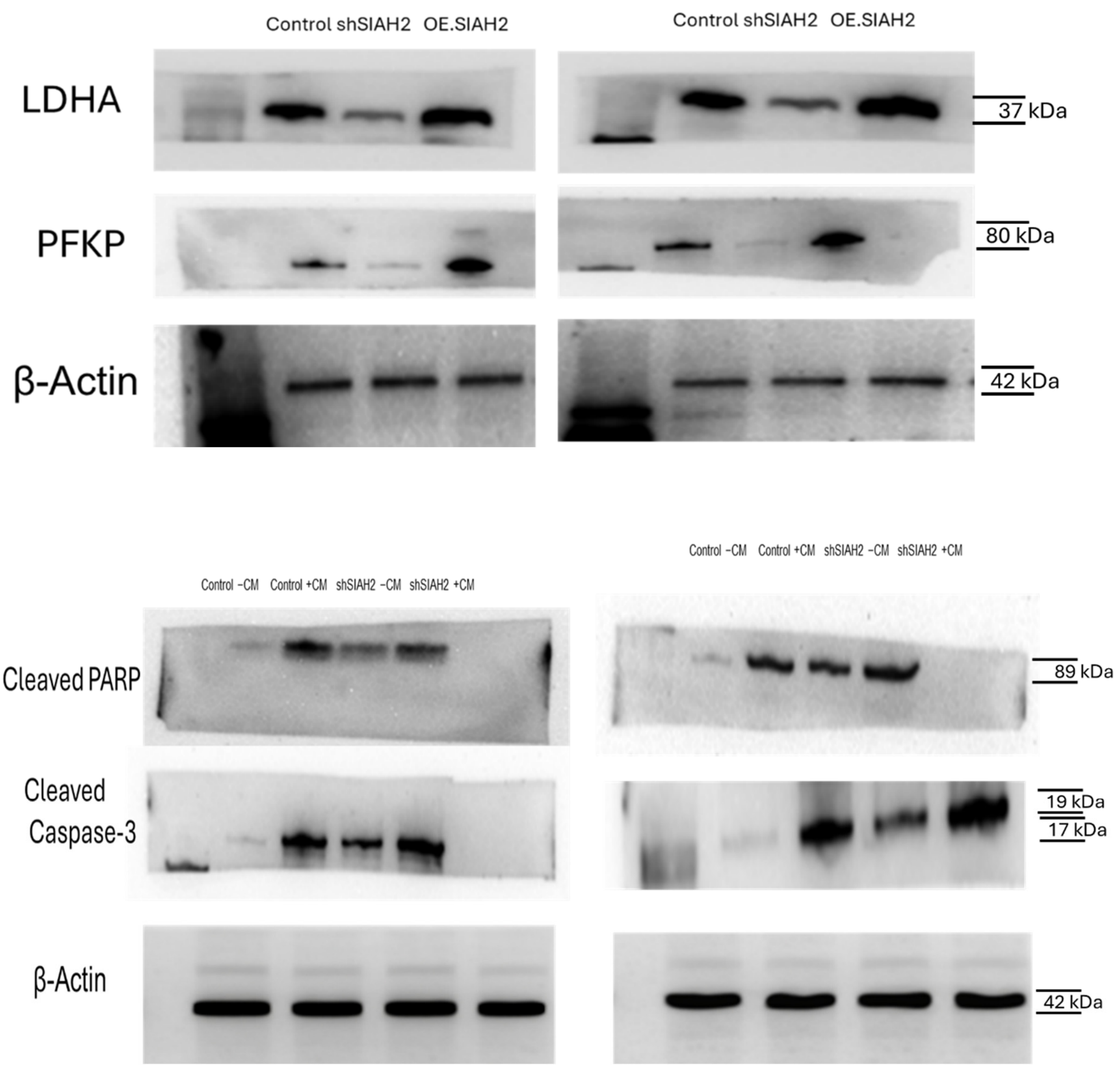

Supplementary Figure S4: Raw uncropped western blot image for supplementary **Figure S1 A-B**.

Supplementary Table S1: List of antibodies used for this study.

Target Protein	Antibody Type / Clone	Host	Company	Catalog No.	Application	Dilution	Expected MW (kDa)
**SIAH2**	Polyclonal	Rabbit	Proteintech	12651-1-AP	WB	1:1000	~34–36
**WNK1**	Polyclonal	Rabbit	Proteintech	28357-1-AP	WB	1:2000	~250–260
**HIF-1α**	Monoclonal	Mouse	Proteintech	20960-1-AP	WB	1:2000	~120
**LDHA**	Monoclonal (C4B5)	Rabbit	Cell Signaling Technology	#2012	WB	1:1000	~36–37
**PFKP**	Polyclonal	Rabbit	Proteintech	13389-1-AP	WB	1:2000	~85–90
**Cleaved PARP (Asp214)**	Polyclonal	Rabbit	Cell Signaling Technology	#9541	WB	1:1000	~89
**Cleaved Caspase-3 (Asp175)**	Polyclonal	Rabbit	Cell Signaling Technology	#9661	WB	1:1000	~17–19
**β-Actin**	Monocolnal	Rabbit	Cell Signaling Technology	#4970	WB	1:1000	~42-45
**Anti-Rabbit IgG (HRP)**	Secondary	Goat	Jackson ImmunoResearch	111-035-144	WB	1:1000	—
